# Characterization of the complete chloroplast genome of *Rhododendron henanense* subsp. *lingbaoense* Fang

**DOI:** 10.1080/23802359.2021.1994898

**Published:** 2021-11-03

**Authors:** Xiao-Jun Zhou, Meng-Jiao Wei, Kai Zhang, Jun-Wang Han, Hai-Liang Wang, Shuai-Wei Dong

**Affiliations:** a College of Life Science, Luoyang Normal University, Luoyang, China; b Henan Xiaoqinling National Nature Reserve Management Bureau, Sanmenxia, China

**Keywords:** *Rhododendron henanense* subsp. *lingbaoense*, chloroplast genome, phylogeny

## Abstract

*Rhododendron henanense* subsp. *lingbaoense* is endemic in China. The cpDNA of *R. henanense* subsp. *lingbaoense* is a typical quadripartite structure with a length of 208,015 bp, including a large single-copy region of 110,593 bp and a small single-copy region of 2606 bp separated by a pair of identical inverted repeat regions of 47,408 bp each. The chloroplast genome contains 119 genes, including 86 protein-coding genes, four ribosomal RNA genes, and 29 transfer RNA genes. The phylogenetic analysis of *R. henanense* subsp. *lingbaoense* showed a relatively close relationship with *Rhododendron delavayi*.

Rhododendron is the collective name of *Rhododendron* (Ericaceae) plants and is one of the top 10 famous flowers in China. In addition to its high ornamental value, rhododendron has the functions of ecological protection, medicinal use, and scientific research (Liang et al. 2016; Li et al. 2018). There are abundant rhododendron germplasm resources in China. However, some unattended wild species with small populations have been extinct or are on the verge of extinction due to the narrow habitats and severe anthropogenic interference (Ma et al. 2014; Liu et al. 2020). *Rhododendron henanense* subsp. *lingbaoense* belongs to the Subgen. *Hymenanthes* and the Subsect. *Campylocarpa* (Fang 1983). It is a key protected plant in Henan Province and only distributed in Henan Xiaoqinling National Nature Reserve at the border of Henan and Shaanxi (altitude 2000 m) (Zhou et al. 2019). Due to its large flower shape and cluster growth, *R. henanense* subsp. *lingbaoense* not only has high landscape utilization value, but also has scientific research value and natural heritage value as an endemic species (Weng et al. 2012). In this study, we sequenced the complete chloroplast genome of *R. henanense* subsp. *lingbaoense* and analyzed its phylogenetic relationship by using complete chloroplast genomes.

The sample of *R. henanense* subsp. *lingbaoense* was collected from Xiaoqinling National Nature Reserve, Lingbao, China (34°25′12.37″N, 110°28′45.19″E). The herbarium vouchers of the plants used in this study are deposited in the Luoyang Normal University Specimen Museum under accession number BOT20200615 (Xiao-Jun Zhou, Email: lynubio@126.com). Genomic DNA was extracted from the leaves using Plant DNAextraction kit (Tiangen, Beijing, China). DNA samples that passed the quality test were sonicated into fragments with an average length of 350 bp by using CovarisM220. After purification, end-terminal repair, 3′-A addition, and adapter ligation were conducted. Sequencing libraries were constructed after PCR amplification and were sequenced using the Illumina Hiseq and PacBio Sequel platforms. The sequencing data were assembled and corrected by ABySS v2.0.2 (http://www.bcgsc.ca/platform/bioinfo/software/abyss) and GapCloser v1.12 software (Luo et al. 2012). CPGAVAS2, RNAmmer-1.2, and tRNAscan-SE v1.3.1 software were used to predict the gene structures (Lagesen et al. 2007; Chan and Lowe 2019; Shi et al. 2019).

The complete chloroplast genome of *R. henanense* subsp. *lingbaoense* is 208,015 bp in length and contains a small single-copy (SSC) of 2606 bp, a large single-copy (LSC) of 110,593 bp, and two inverted repeat (IR) regions of 47,408 bp each (GenBank accession no. MT239363). Its GC content was 35.81%. There were 119 genes, which include 86 protein-coding genes, 29 tRNA, and four rRNA. In total, 18 genes contain one intron, and two genes (*rps12*and *ycf3*) contain two introns. A total of 351 simple sequence repeats (SSRs) were identified in the *R. henanense* subsp. *lingbaoense* chloroplast genome. Among these SSRs, the mononucleotide was the most abundant SSR marker, accounting for 69.80% (245) of the total SSR markers, which was followed by tri- (68, 19.37%), di- (18, 5.13%), tetra- (16, 4.56%), penta- (3, 0.85%), and hexanucleotide (1, 0.28%) SSRs.

The phylogenetic relationships of *R. henanense* subsp. *lingbaoense* were estimated using the maximum-likelihood method in PhyML v3.0 with the LG substitution model (http://www.atgc-montpellier.fr/phyml/). The phylogenetic tree showed that *R. henanense* subsp. *lingbaoense* is relatively closely related to the *R. delavayi* (Figure 1). The *Rhododendron* plants are in a stable monophyletic branch and form sister clades with other groups of the Ericaceous (*Vaccinium* and *Gaultheria*).

**Figure 1.**
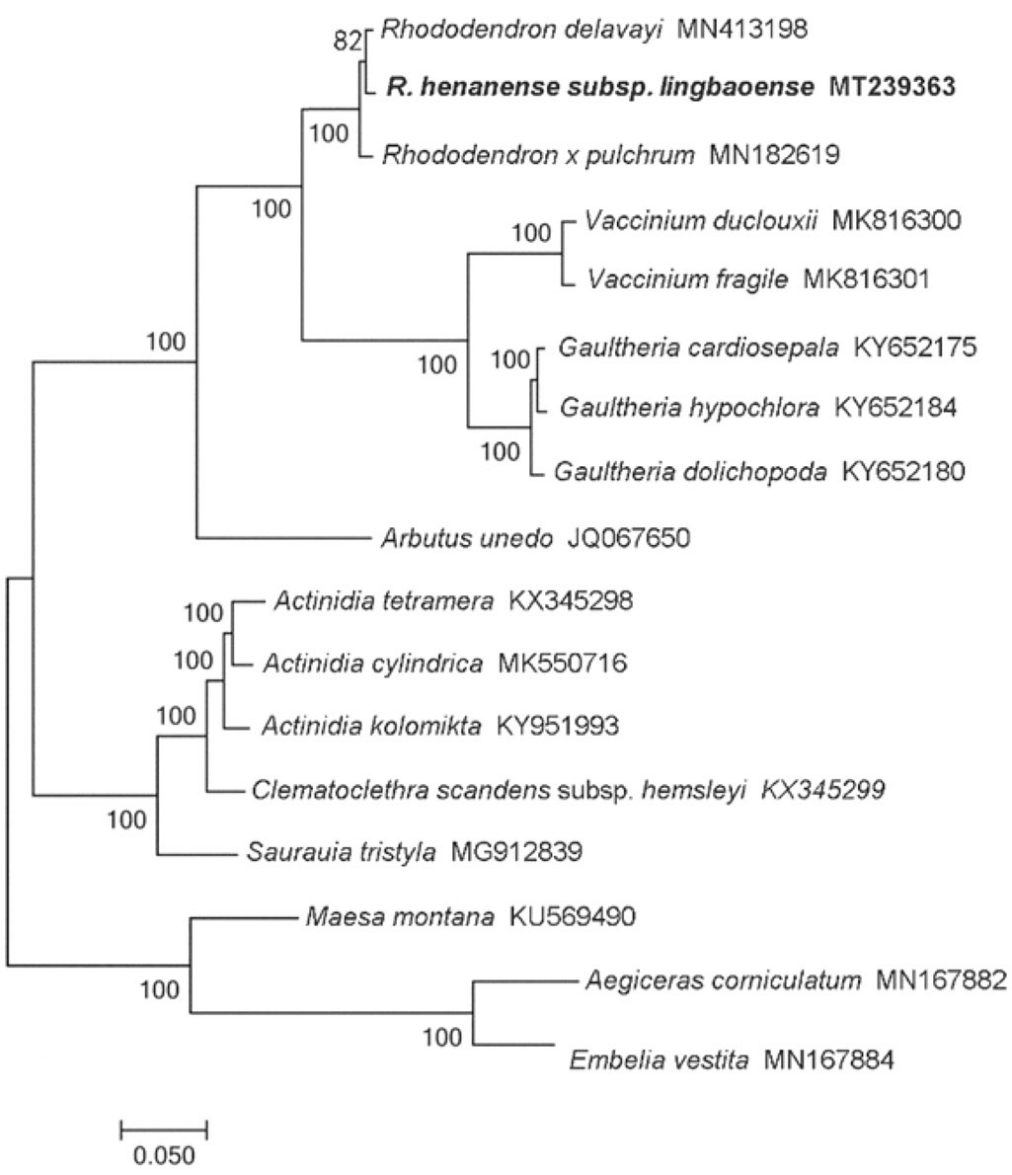
Maximum-likelihood phylogenetic tree for *R. henanense* subsp. *lingbaoense* based on 17 complete chloroplast genomes.

## Data Availability

The complete chloroplast genome sequences of *R. henanense* subsp. *lingbaoense* are openly available in GenBank of NCBI at (https://www.ncbi.nlm.nih.gov/ under the accession number MT239363. The associated BioProject, SRA, and Bio-Sample numbers are PRJNA611883, SRX9089912, and SAMN15794008, respectively.
